# High-dose influenza vaccination and mortality among predominantly male, white, senior veterans, United States, 2012/13 to 2014/15

**DOI:** 10.2807/1560-7917.ES.2020.25.19.1900401

**Published:** 2020-05-14

**Authors:** Yinong Young-Xu, Julia Thornton Snider, Salaheddin M Mahmud, Ellyn M Russo, Robertus Van Aalst, Edward W Thommes, Jason KH Lee, Ayman Chit

**Affiliations:** 1 Clinical Epidemiology Program, Veterans Affairs Medical Center, White River Junction, United States; 2 Department of Psychiatry, Geisel School of Medicine at Dartmouth, Hanover, United States; 3 Precision Health Economics, Oakland, United States; 4 Department of Community Health Sciences, College of Medicine, University of Manitoba, Winnipeg, Canada; 5 George and Fay Yee Center for Healthcare Innovation, University of Manitoba/Winnipeg Regional Health Authority, Winnipeg, Canada; 6 Sanofi Pasteur, Swiftwater, United States; 7 Faculty of Medical Sciences, University of Groningen, Groningen, the Netherlands; 8 Department of Mathematics and Statistics, University of Guelph, Guelph, Canada; 9 Leslie Dan School of Pharmacy, University of Toronto, Toronto, Canada; 10 Sanofi Pasteur, Toronto, Canada

**Keywords:** Influenza vaccines, influenza, high-dose influenza vaccine, vaccine effectiveness, veterans, mortality

## Abstract

**Introduction:**

It is unclear whether high-dose influenza vaccine (HD) is more effective at reducing mortality among seniors.

**Aim:**

This study aimed to evaluate the relative vaccine effectiveness (rVE) of HD.

**Methods:**

We linked electronic medical record databases in the Veterans Health Administration (VHA) and Medicare administrative files to examine the rVE of HD vs standard-dose influenza vaccines (SD) in preventing influenza/pneumonia-associated and cardiorespiratory mortality among VHA-enrolled veterans 65 years or older during the 2012/13, 2013/14 and 2014/15 influenza seasons. A multivariable Cox proportional hazards model was performed on matched recipients of HD vs SD, based on vaccination time, location, age, sex, ethnicity and VHA priority level.

**Results:**

Among 569,552 person-seasons of observation, 207,574 (36%) were HD recipients and 361,978 (64%) were SD recipients, predominantly male (99%) and white (82%). Pooling findings from all three seasons, the adjusted rVE estimate of HD vs SD during the high influenza periods was 42% (95% confidence interval (CI): 24–59) against influenza/pneumonia-associated mortality and 27% (95% CI: 23–32) against cardiorespiratory mortality. Residual confounding was evident in both early and late influenza periods despite matching and multivariable adjustment. Excluding individuals with high 1-year predicted mortality at baseline reduced the residual confounding and yielded rVE of 36% (95% CI: 10–62) and 25% (95% CI: 12–38) against influenza/pneumonia-associated and cardiorespiratory mortality, respectively. These were confirmed by results from two-stage residual inclusion estimations.

**Discussion:**

The HD was associated with a lower risk of influenza/pneumonia-associated and cardiorespiratory death in men during the high influenza period.

## Introduction

Seasonal influenza epidemics result in substantial health burden. Among the 9 million veterans under the care of the United States (US) Veterans Health Administration (VHA), nearly 3,800 are estimated to die annually from respiratory and circulatory complications associated with seasonal influenza infections [1-4]. The clinical risk for hospitalisation and death is highest among persons aged 65 years or older (hereinafter referred to as seniors) because of frailty and immunosenescence [5,6]. In 2009, the US Food and Drug Administration licensed an injectable high-dose inactivated trivalent influenza vaccine (HD) (Fluzone High-Dose, Sanofi Pasteur, Swiftwater, US). The HD contains four times more influenza haemagglutinin antigen than standard-dose influenza vaccines (SD) (60 μg vs 15 μg per strain) and is designed to provide improved protection in seniors.

Beginning with the 2010/11 season, the VHA began to introduce the HD across its medical centres, largely relying on individual facilities to decide the volume to order and on physicians to decide the recipients of the specific type of vaccine. While the majority of VHA senior patients still receive SD, the proportion of HD recipients has been steadily rising, from 3% during 2010/11 to ca 11% during 2014/15 (data not shown). Senior VHA patients can also obtain influenza vaccination using non-VHA insurance coverage, such as that provided by Centers for Medicare and Medicaid Services (Medicare), once they become eligible.

Clinical trials have shown that HD was 7% (95% confidence interval (CI): 0.5–12.8) and 8% (95% CI: 0.3–14) more effective than SD in preventing all-cause hospital admissions in ambulatory and nursing home seniors, respectively [7,8]. The advantage of HD in preventing hospitalisations has also been confirmed by several observational studies [9-13]. However, it is unclear whether HD is more effective at reducing mortality among seniors, especially given the ongoing debate surrounding the scale of mortality attributable to influenza and the effectiveness of influenza vaccines among the elderly. Although a number of HD studies have reported mortality outcomes [7-10], only one study has investigated this question as a primary objective: Shay et al. found that HD vaccination was 24% (95% CI: 6–42) more effective in preventing mortality after influenza hospital admission [12]. Nevertheless, potential confounding because of indication associated with ‘frailty’ bias remains to be examined and mitigated [14,15]. We examined the relative vaccine effectiveness (rVE) of HD compared with SD against mortality outcomes among VHA veterans during the 2012/13, 2013/14 and 2014/15 influenza seasons.

## Methods

### Design and data sources

The VHA is the single largest integrated healthcare system in the US and provides clinical care to more than 9 million military veterans at more than 170 medical centres and ca 1,400 community-based outpatient clinics. We obtained de-identified VHA electronic medical records (EMR) data and administrative health records for VHA enrolees from Medicare fee-for-service files. These records supplement the VHA database as many VHA patients seek healthcare outside the VHA system once they turn 65 and qualify for such additional benefits. We obtained vital status from the VHA vital status files and death certificate data from the National Death Index (NDI) through the Centre of Excellence for Suicide Prevention Joint Department of Veterans Affairs and Department of Defence Suicide Data Repository – NDI (extracted: 22 January 2019) [16].

Using these data sources, a retrospective cohort study was conducted to compare the risk of mortality among HD and SD recipients for the 2012/13, 2013/14 and 2014/15 seasons. Two causes of death were examined: (i) influenza- or pneumonia-associated and (ii) cardiorespiratory, as these were likely to be impacted by influenza vaccination [10]. Causes of death were classified using the International Classification of Diseases, 10th revision (ICD-10) [17].

### Ethical statement

This study was approved by the Veteran’s Institutional Review Board of Northern New England at the White River Junction VHA Medical Center (No. 903343). All study procedures were carried out in compliance with federal and institutional ethical guidelines. The requirement to obtain informed consent from study participants was waived as there was no more than a minimal risk to the privacy of individuals.

### Study population and influenza vaccination

The study population included all VHA enrolees who turned 66 years or older by 1 July for each of the influenza seasons and maintained their enrolment until the end of the season (30 June of the following year) or until death, whichever occurred earlier. Influenza vaccination was identified using current procedural terminology (CPT) codes (SD CPT codes: 90655–90659 and Q2034-Q2039; HD CPT code: 90662). We included veterans who received either an HD or SD and excluded any who did not have a record of vaccination or received more than one influenza vaccine in the same season to ensure valid comparison.

### Influenza activity periods

**Figure 1:**
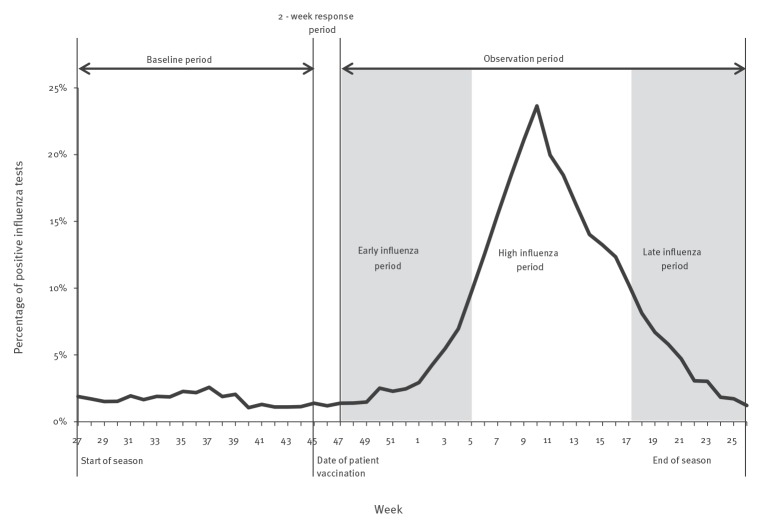
Schematic overview of influenza season and study periods, United States, 2012/13–2014/15

For each Centers for Disease Control and Prevention (CDC) multi-state reporting region [18], we divided each season into three periods of potential influenza activity, following methods employed in previous research [19]. We used weekly reports of the percentage of positive influenza tests among all influenza tests performed in each region to define (i) *high influenza period* as the time between the first and last occurrences of 2 consecutive weeks with at least 10% influenza positivity, (ii) *early influenza period* as the time from 1 September to the start of the high influenza period and (iii) *late influenza period* as time from the end of the high influenza period to the end of June (Figure 1).

### Baseline characteristics

For each study subject, the baseline period began at the end of each previous season in week 27 (beginning of July) and ended at his/her influenza vaccination date. Characteristics measured during the baseline period included demographics, comorbidities, and healthcare utilisation. Demographics comprised age, sex, ethnicity, geographic region and priority rating of VHA care (as a proxy for socioeconomic status because it is partially based on income and the capacity for gainful employment) [20].

Comorbidities were defined according to an adaptation of Deyo-Charlson comorbidity score [21] using diagnosis codes captured during hospital and ambulatory visits. As a proxy measure for frailty, we used the care assessment need (CAN) score developed specifically to predict hospitalisation within 1 year among VHA patients. In addition to incorporating the medical conditions used in the Charlson and Elixhauser scores [22], CAN includes sociodemographic characteristics, the prior year’s levels of healthcare utilisation (e.g. number of primary care, non-emergency department outpatient visits), medication use and laboratory test results [23]. We used the maximum CAN score in the 4 weeks before vaccination. Healthcare utilisation was measured as the number of all-cause hospitalisations.

### Matching

Each HD recipient was matched to at least one, and at most two, residents of the same VHA facility who received an SD within the same week. This process addressed temporal and geographical factors possibly associated with access to HD and influenza exposure (i.e. influenza outbreak activity). In addition, these HD and SD recipients were matched on all demographic variables including age group (65–74, 75–84 and ≥ 85 years), sex, ethnicity (white vs other) and VHA priority rating (high vs low). All analyses were performed on the matched populations.

### Statistical analysis

We used standardised mean difference (SMD) as a measure of statistical differences between two groups. SMD was calculated by dividing the difference in mean outcome between groups by the pooled standard deviation of the two groups. The absolute value of this division is then multiplied by 100, with a value greater than 10 denoting statistical significance [24]. Cox proportional hazards modelling was used to estimate the hazard ratios (HR) and 95% CI for the association between receipt of the HD and mortality separately for each outcome and influenza activity period. Within each influenza period, follow-up time began on the index date, defined as 2 weeks following vaccination, or the beginning of each influenza period, whichever came last. This was done because in primed healthy adults, the peak serum antibody levels are typically observed 2 weeks post-vaccination [25]. We excluded study subjects who received vaccination within 15 days of the end of each influenza period to allow for at least 1 day of follow-up. The observation period ended on the date of disenrollment from either VHA or Medicare, end of each of the three influenza season periods or date of death, whichever occurred first. For example, if a patient was vaccinated on 1 October, then his/her follow-up time for the early influenza period began on 15 October. If he/she died from an influenza/pneumonia-associated cause on 1 December, his/her follow-up time would end then, regardless of whether the high influenza period in his/her region had begun. The models adjusted for all baseline comorbidities and healthcare utilisation and adjusted for demographics through matching.

We also conducted analyses with mortality as a binary outcome. This was achieved using a two-stage residual inclusion model, also known as control functions approach [26], to account for potential confounding from measured and unmeasured variables. The rVE was calculated as (1−HR) × 100%. All tests were two-tailed, and 0.05 was the level of statistical significance. We performed statistical analyses using Stata 15 (StataCorp, College Station, TX).

To further quantify the presence of confounding by indication due to frailty, we used a logistic regression model to predict mortality for all cohort members during each influenza season. We then re-ran the fully adjusted Cox proportional hazard model on the subpopulation that excluded individuals at higher levels of illness severity, as measured by predicted one-year mortality [19]. We estimated rVE for each season separately, and then pooled the results after statistically examining heterogeneity caused by inter-season variability.

## Results

**Figure 2:**
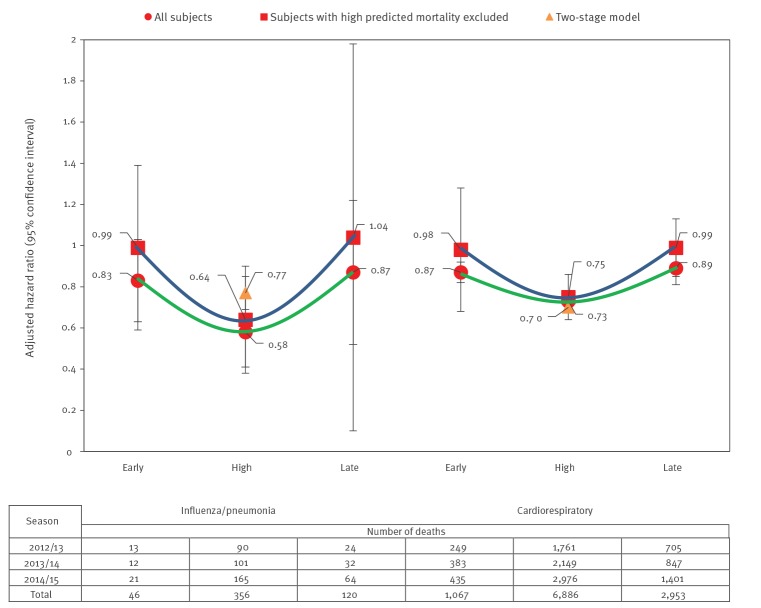
Hazard ratios pooled over three influenza seasons and number of deaths by cause and influenza period, among predominantly male, white, senior veterans, United States, 2012/13–2014/15 (n = 569,552)

We included 569,552 person-seasons of observation where 207,574 (36%) were among HD recipients and 361,978 (64%) were among SD recipients during the three seasons; 99% were male and 80% were of non-Hispanic white origin (Table 1). We matched 49,950, 65,267 and 92,357 HD recipients to 89,700, 117,518 and 154,760 SD recipients for the 2012/13, 2013/14 and 2014/15 season, respectively. Matched HD or SD recipients were similar with respect to baseline covariates as well as CAN score. For the matched cohorts, we observed 127, 145 and 250 influenza/pneumonia-associated deaths and 2,715, 3,379 and 4,812 cardiorespiratory deaths for the 2012/13, 2013/14 and 2014/15 season, respectively (Figure 2).

**Table 1:** Baseline characteristics after matching by standard dose vs high-dose influenza vaccination among predominantly male, white, senior veterans , United States, 2012/13–2014/15 (n = 569,552)

Season		2012/13			2013/14			2014/15		
Influenza vaccine		SD	HD	SMD^a^	SD	HD	SMD^a^	SD	HD	SMD^a^
Study population		89,700	49,950		117,518	65,267		154,760	92,357	
Sex	Female	1%	1%	1	1%	1%	1	1%	1%	1
	Male	99%	99%	0	99%	99%	1	99%	99%	1
Ethnicity	White	80%	79%	3	82%	81%	2	83%	83%	2
	African-American	8%	10%	4	8%	7%	3	8%	7%	3
	Hispanic	8%	8%	1	7%	8%	6	6%	7%	5
	Other	2%	2%	1	2%	2%	1	2%	2%	1
	Unknown	1%	1%	0	1%	1%	0	1%	1%	0
Age group (years)	65–74	37%	38%	2	40%	41%	2	44%	45%	1
	75–84	43%	42%	2	40%	40%	1	37%	37%	1
	≥ 85	20%	20%	1	20%	19%	1	19%	19%	1
Priority	High priority	38%	39%	1	40%	40%	0	42%	42%	1
	Low priority	62%	61%	1	60%	60%	0	58%	58%	1
HHS region	1	5%	4%	2	6%	5%	1	8%	7%	2
	2	9%	9%	2	9%	9%	1	12%	11%	2
	3	11%	11%	2	11%	11%	1	11%	13%	6
	4	28%	28%	0	30%	30%	1	28%	27%	2
	5	14%	11%	8	12%	10%	5	11%	10%	5
	6	9%	11%	4	9%	10%	1	7%	7%	0
	7	11%	14%	9	9%	12%	9	8%	10%	8
	8	3%	3%	1	3%	3%	1	4%	3%	2
	9	7%	7%	2	9%	8%	1	10%	10%	0
	10	3%	3%	0	2%	2%	1	2%	2%	2
Morbidity	Malignancy	16%	18%	5	16%	18%	7	16%	18%	6
	Metastatic solid tumour	1%	1%	0	1%	1%	0	1%	1%	1
	Congestive heart failure	12%	12%	2	11%	12%	2	11%	12%	1
	Chronic pulmonary disease	19%	20%	3	19%	20%	5	19%	20%	2
	Cerebrovascular disease	10%	11%	3	9%	10%	3	9%	10%	3
	Dementia	2%	2%	0	2%	2%	1	2%	2%	0
	Diabetes with complications	10%	11%	3	10%	11%	3	11%	12%	3
	Diabetes without chronic complications	39%	39%	1	39%	39%	1	39%	39%	0
	HIV/AIDS	0.2%	0.2%	1	0.2%	0.2%	0	0%	0%	1
	Mild liver disease	1%	1%	0	2%	2%	0	2%	2%	1
	Moderate/severe liver disease	0.2%	0.2%	0	0.2%	0.2%	1	0%	0%	0
	Myocardial infarction	3%	3%	0	3%	3%	2	3%	3%	1
	Hemiplegia/paraplegia	1%	1%	0	1%	1%	1	1%	1%	1
	Peptic ulcer disease	1%	1%	1	1%	1%	1	1%	1%	1
	Peripheral vascular disease	13%	14%	4	12%	14%	5	12%	14%	6
	Rheumatoid disease	2%	2%	2	2%	2%	2	2%	2%	2
	Renal disease	13%	13%	0	13%	13%	2	13%	13%	1
Continuous variables^b^	Age	74.5	74.9	5	76.9	77.1	1	76.6	76.7	2
	Number of hospitalisations	0.19	0.18	2	0.13	0.11	3	0.14	0.12	5
	VHA care assessment need score	0.05	0.04	3	0.054	0.047	9	0.05	0.04	9

HD: high-dose influenza vaccine; HHS: Health and Human Services; VHA: Veterans Health Administration; SD: standard-dose influenza vaccine; SMD: standardised mean difference.

^a^ An SMD less than 10 in absolute value suggests no important difference between the two cohorts [24].

^b^ Continuous variables reported as mean.

During the high influenza periods, rVE ((1−HR) × 100%) for influenza/pneumonia-associated mortality was 23% (95% CI: −83 to 67), 32% (95% CI: −17 to 60) and 47% (95% CI: 21–65) for seasons 2012/13, 2013/14 and 2014/15, respectively, with a pooled rVE of 42% (95% CI: 24–59), presented in Table 2. For cardiorespiratory deaths, the rVE were 23% (95% CI: 13–32), 28% (95% CI: 19–35), and 30% (95% CI: 23–36) for the three study seasons, respectively, with a pooled rVE of 27% (95% CI: 23–32).

**Table 2:** Relative vaccine effectiveness of high-dose vs standard dose influenza vaccination and mortality by influenza period, matched and adjusted using Cox proportional hazards model, among predominantly male, white, senior veterans, United States, 2012/13–2014/15 (n = 569,552)

Population	Season	Influenza/pneumonia cause of death			Cardiorespiratory cause of death		
		Influenza period					
		Early	High	Late	Early	High	Late
		rVE % (95% CI)			rVE % (95% CI)		
All subjects	2012/13	0 (−56–36)	23 (−83 to 67)	4 (−143 to 62)	5 (−41 to 37)	23 (13 to 32)	3 (−39 to 32)
	2013/14	10 (−88 to 57)	32 (−17 to 60)	13 (−86 to 59)	7 (−8 to 20)	28 (19 to 35)	9 (−5 to 22)
	2014/15	23 (−5 to 43)	47 (21 to 65)	16 (−46 to 52)	14 (8 to 20)	30 (23 to 36)	13 (2 to 22)
	*Pooled*	17 (−3 to 37)	42 (24 to 59)	13 (−22 to 48)	13 (7 to 18)	27 (23 to 32)	11 (3 to 19)
Excluding subjects with greater than 5% predicted mortality	2012/13	−1 (−60 to 36)	22 (−79 to 73)	−5 (−696 to 86)	−4 (−130 to 53)	24 (−18 to 51)	−3 (−34 to 21)
	2013/14	−2 (−124 to 53)	37 (−9 to 64)	1 (−119 to 55)	2 (−30 to 26)	21 (4 to 35)	0 (−28 to 22)
	2014/15	6 (−165 to 67)	41 (9 to 55)	−8 (−389 to 76)	8 (−18 to 28)	31 (19 to 41)	7 (−13 to 24)
	*Pooled*	1 (−44 to 46)	36 (10 to 62)	−4 (−98 to 89)	2 (−28 to 32)	25 (12 to 38)	1 (−12 to 15)

CI: confidence interval; rVE: relative vaccine effectiveness.

During the early influenza periods, rVE (1−HR) for influenza/pneumonia-associated mortality was 0% (95% CI: −56 to 32), 10% (95% CI: −88 to 57) and 23% (95% CI: −5 to 43) for seasons 2012/13, 2013/14 and 2014/15, respectively, with a pooled rVE of 17% (95% CI: −3 to 37). For cardiorespiratory deaths, rVE was 5% (95% CI: −41 to 37), 7% (95% CI: −8 to 20) and 14% (95% CI: 8–20) for the three study seasons, respectively, with a pooled rVE of 13% (95% CI: 7–18).

During the late influenza periods, rVE (1−HR) for influenza/pneumonia-associated mortality was 4% (95% CI: −143 to 62), 13% (95% CI: −86% to 59%) and 16% (95% CI: −46 to 52) for seasons 2012/13, 2013/14 and 2014/15, respectively, with a pooled rVE of 13% (95% CI: −22 to 48). For cardiorespiratory deaths, rVE was 3% (95% CI: −39 to 32), 9% (95% CI: −5 to 22) and 13% (95% CI: 2–22) for the three study seasons, respectively, with a pooled rVE of 11% (95% CI: 3–19).

One-year mortality was accurately predicted (C-statistic = 0.84) using a logistic regression model. Excluding individuals with greater than 5% predicted mortality (ca 21% of the cohort) had a considerable impact on the HR estimates (Figure 2). During the early influenza periods, pooled rVE for influenza/pneumonia-associated mortality was 1% (95% CI: −44 to 46) and 2% (95% CI: −28 to 32) for cardiorespiratory deaths, noticeably closer to no effect (i.e. rVE = 0%) compared with the analysis including all cohort members (Table 2). The estimates for the late influenza periods also moved closer to the null when individuals at high predicted probabilities of mortality were excluded; pooled rVE were −4% (95% CI: −98 to 89) and 1% (95% CI: −12 to 15) for influenza/pneumonia-associated and cardiorespiratory mortality, respectively. Excluding individuals with high 1-year predicted mortality at baseline reduced the residual confounding and yielded rVE of 36% (95% CI: 10–62) and 25% (95% CI: 12–38) against influenza/pneumonia-associated mortality and cardiorespiratory mortality, respectively, during the high influenza period.

Confounding by indication was further studied with a two-stage residual inclusion model. Pooling findings from all three seasons, the adjusted rVE estimate of HD vs SD during the high influenza periods was 23% (95% CI: 17–28) against influenza/pneumonia-associated mortality and 30% (95% CI: 23–38) against cardiorespiratory mortality (Table 3). The estimates differed by season, but with overlapping CI. This was more evident for influenza/pneumonia-associated mortality: the rVE was 29% (95% CI: 21–36), 11% (95% CI: −1 to 22) and 21% (95% CI: 11–30) for the three seasons, respectively. Finally, we estimated adjusted rVE during the baseline (summer) period against all-cause hospitalisations to assess confounding by indication. The rVE during the baseline period were close to the null: −7% (95% CI: −21–5), −6% (95% CI: −11 to −2) and 5% (95% CI: 2–8) for the three seasons, respectively, for a pooled estimate of −1% (95% CI: −5–2) (Table 3).

**Table 3:** Relative vaccine effectiveness of high-dose vs standard dose influenza vaccination and mortality during high influenza period, matched and adjusted using two-stage residual inclusion model, among predominantly male, white, senior veterans, United States, 2012/13–2014/15 (n = 569,552)

Season	Influenza/pneumonia cause of death	Cardiorespiratory cause of death	All-cause hospitalisations (baseline)
	rVE % (95% CI)	rVE % (95% CI)	rVE % (95% CI)
2012/13	29 (21 to 36)	26 (2 to 44)	−7 (−21 to 5)
2013/14	11 (−1 to 22)	29 (27 to 30)	−6 (−11 to −2)
2014/15	21 (11 to 30)	31 (30 to 32)	5 (2 to 8)
*Pooled*	23 (17 to 28)	30 (23 to 38)	−1 (−5 to 2)

CI: confidence interval; rVE: relative vaccine effectiveness.

## Discussion

In our analysis of the 2012/13, 2013/14 and 2014/15 influenza seasons, we found receiving HD among a population of predominantly male, white, senior veterans to be associated with an additional reduction in mortality as compared with receiving SD. Although only a portion of all winter deaths can be attributed to influenza [27], the additional 36% (95% CI: 10–62) reduction in influenza/pneumonia-associated mortality and 25% (95% CI: 12–38) reduction in cardiorespiratory mortality for HD vs SD present substantial impact as nearly 3,800 VHA patients die annually from respiratory and circulatory complications associated with seasonal influenza infections [1].

An HR of 1 implies a null effect (i.e. no difference in vaccine effectiveness). Thus, we expected to see HR furthest from the null, indicating largest relative vaccine effect, for the periods when influenza is in high circulation and more likely to trigger the largest proportion of deaths. For all seasons, and for both influenza/pneumonia-associated and cardiorespiratory mortality, we observed the same pattern: The HR were the furthest from the null during the high influenza period, while HR were much closer to the null during the early and late influenza periods. This created a U-shape pattern, suggesting statistically significant rVE during the high activity periods. During the early and late influenza periods, most rVE estimates associated with either cardiorespiratory or influenza/pneumonia-associated mortality displayed evidence of differential impact outside the high activity periods, albeit smaller. For example, the rVE for cardiorespiratory mortality were both statistically significant for the 2014/15 season, 14% (95% CI: 8–20) in the early period and 13% (95% CI: 2–22) in the late period, if not controlled for residual confounding. During the early and late influenza periods, when influenza viruses were not circulating or circulating at a low level, these significant rVE could indicate the presence of residual confounding after matching and multivariable survival analysis. The appearance and confirmation of residual confounding during these periods are not new. Campitelli et al., among others, have found similar bias in their study of influenza vaccines’ impact on mortality [19]. However, this bias cannot explain away all the observed mortality benefit associated with HD during the high influenza periods, as the magnitudes of the rVE for the early and late influenza periods were half to one third of the rVE during the high influenza period. Furthermore, excluding individuals with high 1-year predicted mortality at baseline resulted in rVE much closer to the null, indicating reduced levels of residual confounding. Receipt of SD was more prevalent than HD among veterans with high 1-year predicted mortality (Supplementary Table 1), which impacted the estimate of rVE. Before this exclusion, the average 1-year predicted mortality for HD and SD recipients was 3.3% and 4.2%, respectively, with a not statistically significant SMD of 9. This suggests a successful balance by matching of this surrogate measure of frailty. Nevertheless, the exclusion approach appeared to have a larger impact than matching, which could suggest that HD conferred better protection relative to SD for the frailest veterans. Finally, it was reassuring that almost no residual confounding was observed during the non-influenza circulation period (summer) against all-cause hospitalisations.

The relative effectiveness between HD and SD was estimated during three seasons with varying circulating strains and vaccine efficacy. While influenza A(H3N2) viruses predominated in both the 2012/13 [28] and 2014/15 [29] seasons, 2013/14 [30] was the first influenza A(H1N1)pdm09–predominant season since 2009. Nevertheless, VE were similar for both the 2012/13 (49%; 95 CI: 43–55) and 2013/14 season (52%; 95 CI: 44–59) but dramatically lower for the 2014/15 season, at an overall effectiveness of 19% (95 CI: 10–27) [31]. The combination of low VE and predominant influenza A(H3N2) viruses, which are associated with higher rates of influenza-associated hospitalisations among the elderly, may have contributed to the highest recorded rate of laboratory-confirmed, influenza-associated hospitalisations in the US, at 319.2 per 100,000 population in the 2014/15 season. This rate exceeded the previously highest record of 183.2 per 100,000 for the 2012/13 season by 74% [29]. Given the varying rVE, VE and circulating viruses, it would be interesting to examine the association among the three. Unfortunately, including these variables in the model would add greater complexity and require additional seasons and data. Moreover, some veterans’ vaccinations were not recorded in the VHA or CMS databases, resulting in incomplete vaccination data. We believe this incompleteness, and resulting misclassification, could have had a substantial impact on the estimation of VE. To evaluate the VE of each vaccine or to study the impact of repeated vaccinations would also require significant undertaking to avoid misclassifying those without vaccination records as being not vaccinated. To convey clear public health messages, we believe these should be studied carefully in the future.

Our comparative effectiveness analysis of HD and SD vaccination on mortality explores the same question over the same period as Shay et al.’s analysis, but with differences in study populations, healthcare providers, outcome definitions and statistical methods. Nevertheless, our results were consistent with their overall rVE: 24% (95% CI: 6–42) in post-influenza deaths, defined as “*a death occurring in the 30 days following a Medicare claim for an inpatient hospitalisation or an emergency department visit with a diagnosis of influenza.*” [12] Clinical trial data have shown that the VE of HD might be greater for A(H3N2) than for A(H1N1) influenza viruses [9]. This could explain some variation by season in our study, as our rVE were 29% (95% CI: 21–36) for 2012/13 (predominantly A(H3N2)), 11% (95% CI: −1 to 22) for 2013/14 (predominantly A(H1N1)pdm09) and 21% (95% CI: 11–30) for 2014/15 season (predominantly A(H3N2)). The pattern was similar, although less drastic than observed by Shay et al.: 36.4% (95% CI: 9.0–56) for 2012/13 and 2.5% (95% CI: −47 to 35) for 2013/14 season. We focused on the high influenza period while Shay et al. analysed vaccinations that occurred mostly in what we categorised as the early and high influenza periods. However, they defined the period by the proportion of positive tests ≥ 75th percentile for each region/season, which is similar to the 10% positive test rate that we used. Shay et al’s primary method of adjusting for potential confounding, in addition to restricting participation to beneficiaries who were vaccinated in pharmacies, was multivariable adjustment, but they did not conduct matching or additional statistical modelling.

### Strengths

The appropriate analysis and estimation of rVE depends on the correct adjustment for confounding by indication where some of the confounders are unmeasured. In our studies, we attempted three different approaches: (i) the previous event rate ratio method, a type of difference-in-differences analysis [14], (ii) the instrumental variable method, an econometric technique [15] and (iii) the current approach where findings of a vaccine effect during early and late influenza periods could indicate the presence of residual confounding after matching and multivariable analysis. Each approach has strengths and weaknesses that could be amplified or ameliorated with specific study populations, databases or influenza seasons examined. In this study, we experimented with different methods (e.g. two-stage residual inclusion estimation model) and cut-offs (e.g. high 1-year mortality risk) that sometimes resulted in divergent point estimates or wide CI, which could be due to varying sample sizes and statistical assumptions. As we continue to improve on our approaches, we hope that a pattern will emerge from this body of work that could contribute to the scientific inquiry of the rVE of HD and SD.

Moreover, we analysed more than half a million vaccinations and almost 10 thousand deaths. The large sample allowed us to adjust for more confounding variables without compromising statistical power, although not calculated beforehand. We combined data from VHA EMR records and Medicare administrative claims to compile a complete picture of healthcare encounters experienced by our study population. We studied both influenza/pneumonia and cardiorespiratory mortality, using the CDC’s gold standard causes-of-death data [32]. We matched on the location and time of vaccination to better account for temporal and geographical factors associated with influenza disease movement. In addition, we matched on age group, sex, ethnicity and VHA priority rating. This comprehensive matching has been shown to improve the accuracy of estimation, although we would have constructed finer age groups if we had a larger sample size [33].

### Limitations

The CDC’s causes-of-death data lacks standardisation [32]. Since influenza and pneumonia are comparatively rare as causes of death, their designation might be more subjective especially during periods of low influenza activity. Thus, under-diagnosing could lead to variation in assignments of underlying cause of death. The VHA has a unique population: it is more than 90% male, predominantly white, and tends to have greater disease burden than the general US population, thus our finding is not generalisable [34]. Despite combining EMR records from the VHA and administrative claims from Medicare, a small amount of data might still be missing for those enrolled in Medicare Advantage (MA) plans. The proportion that enrolled in MA after vaccination was small (2.5%), and since the most important data to the study were their vaccination status (HD vs SD), mortality outcome and baseline characteristics, the amount of missing data was negligible in the context of our study. Ninety-nine per cent of the vaccines we studied were egg-based with a 1:2 ratio between trivalent HD and trivalent SD in our matched samples. Two-thirds of the trivalent SD were supplied by the same company. Although rVE might differ depending on the specific types of SD (e.g. cell-based, adjuvanted, etc.), we did not have the needed sample size to study the impact of different vaccine types. Additional data on morbidities such as obesity could also have improved measurement on confounding. Finally, VE and rVE are likely to be impacted by the season-dependent match between the vaccine and the predominant circulating strain, as well as by the severity of the influenza season [13].

## Conclusion

Using a combination of approaches, we estimated rVE and mortality in a predominantly male, white, senior VHA patient population. We found that HD was associated with a lower risk of influenza/pneumonia-associated and cardiorespiratory mortality during the high influenza period, an effect that could vary by season.

## Supplementary Data

31000 Supplement
