# Supplementary material for: High-dose influenza vaccination and mortality among predominantly male, white, senior veterans, United States, 2012/13 to 2014/15
Source: Euro Surveill. 2020 May 14;25(19):1900401. doi: 10.2807/1560-7917.ES.2020.25.19.1900401 (PMC7238741; doi:10.2807/1560-7917.ES.2020.25.19.1900401)
Supplement: Supplement [file 19-00401_Supplement.pdf]

Supplementary Table S1. Matching Frequency and Proportion of Subjects with High Mortality Risk

"This supplementary material is hosted by Eurosurveillance as supporting information alongside the article [High-dose influenza vaccination and mortality among predominantly male, white, senior veterans, United States, 2012/13 to 2014/15], on behalf of the authors, who remain responsible for the accuracy and appropriateness of the content. The same standards for ethics, copyright, attributions and permissions as for the article apply. Supplements are not edited by Eurosurveillance and the journal is not responsible for the maintenance of any links or email addresses provided therein."

Season	2012-13	2013-14	2014-15
Matching Frequency			
HD recipients with 1 matched SD recipient	21%	20%	32%
HD recipients with 2 matched SD recipient	79%	80%	68%
Proportion of Subjects with High One-year Predicted Mortality			
SD recipients	20%	25%	24%
HD recipients	14%	17%	18%
